# Effects of a change in recall period on reporting severe symptoms: an analysis of a pragmatic multisite trial

**DOI:** 10.1093/jnci/djae049

**Published:** 2024-03-05

**Authors:** Roshan Paudel, Andrea C Enzinger, Hajime Uno, Christine Cronin, Sandra L Wong, Don S Dizon, Hannah Hazard Jenkins, Jessica Bian, Raymond U Osarogiagbon, Roxanne E Jensen, Sandra A Mitchell, Deborah Schrag, Michael J Hassett

**Affiliations:** Dana-Farber Cancer Institute, Boston, MA, USA; Dana-Farber Cancer Institute, Boston, MA, USA; Dana-Farber Cancer Institute, Boston, MA, USA; Dana-Farber Cancer Institute, Boston, MA, USA; Dartmouth Hitchcock Medical Center, Lebanon, NH, USA; Lifespan Cancer Institute and Brown University, Providence, RI, USA; West Virginia University Cancer Center, Morgantown, WV, USA; Maine Medical Center, Portland, ME, USA; Baptist Medical Center, Memphis, TN, USA; National Cancer Institute, Rockville, MD, USA; National Cancer Institute, Rockville, MD, USA; Memorial Sloan Kettering Cancer Center, New York, NY, USA; Dana-Farber Cancer Institute, Boston, MA, USA

## Abstract

**Background:**

Optimal methods for deploying electronic patient-reported outcomes to manage symptoms in routine oncologic practice remain uncertain. The electronic symptom management (eSyM) program asks chemotherapy and surgery patients to self-report 12 common symptoms regularly. Feedback from nurses and patients led to changing the recall period from the past 7 days to the past 24 hours.

**Methods:**

Using questionnaires submitted during the 16 weeks surrounding the recall period change, we assessed the likelihood of reporting severe or moderate and severe symptoms across 12 common symptoms and separately for the 5 most prevalent symptoms. Interrupted time-series analyses modeled the effects of the change using generalized linear mixed-effects models. Surgery and chemotherapy cohorts were analyzed separately. Study-wide effects were estimated using a meta-analysis method.

**Results:**

In total, 1692 patients from 6 institutions submitted 7823 eSyM assessments during the 16 weeks surrounding the recall period change. Shortening the recall period was associated with lower odds of severe symptom reporting in the surgery cohort (odds ratio* *=* *0.65, 95% confidence interval* *=* *0.46 to 0.93; *P *=* *.02) and lower odds of moderate and severe symptom reporting in the chemotherapy cohort (odds ratio* *=* *0.83, 95% confidence interval* *=* *0.71 to 0.97; *P *=* *.02). Among the most prevalent symptoms, 24-hour recall was associated with a lower rate of reporting postoperative constipation but no differences in reporting rates for other symptoms.

**Conclusion:**

A shorter recall period was associated with a reduction in the proportion of patients reporting moderate-severe symptoms. The optimal recall period may vary depending on whether electronic patient-reported outcomes are collected for active symptom management, as a clinical trial endpoint, or another purpose.

ClinicalTrials.gov ID NCT03850912.

Electronic patient-reported outcomes enable clinical teams to monitor and actively manage patient symptoms remotely, with the potential to improve outcomes, increase patient satisfaction, and affect overall treatment success (1-3). Integration of patient-reported outcomes into electronic health records for active symptom management is gaining traction (4-6). If electronic patient-reported outcomes are to be integrated successfully into routine practice, it is important to consider aspects of electronic patient-reported outcome deployment, including recall period, frequency of symptom reporting, and potential changes to clinic workflows resulting from increased alerts for severe or worsening symptoms (7,8).

In clinical trials, decisions regarding instrument selection (and its associated recall period), frequency, and timing of assessments are guided by considerations that include the study aims, the anticipated pattern of symptomatic adverse events, patient and trialist burden, and the tradeoffs between resources and requirements (9-12). Prior studies of symptom surveillance in clinical trials have used “the past 7 days,” which is the standard recall period for the Patient-Reported Outcomes version of the Common Terminology Criteria for Adverse Events (PRO-CTCAE) instrument (9,13-15). In routine practice, however, a 7-day recall period may not accurately reflect current symptoms because patients may report symptoms that have already resolved, creating alerts that are not timely and lessening clinical actionability.

Prior studies have compared the effects of various recall periods, primarily to establish a reliable time frame to avoid losing any pertinent symptom information while minimizing measurement error and survey fatigue (9,10,14,16). Although these comparisons are well suited to cataloging adverse events in clinical trials, to our knowledge, no studies have compared the reporting of moderate or severe symptoms using a 7-day vs 24-hour recall period in routine practice. We evaluated the effects of a change from a 7-day to a 24-hour recall period on rates of reporting severe symptoms in patients receiving chemotherapy or undergoing surgery in a multicenter, pragmatic, cluster randomized study.

## Methods

### Setting

To improve active symptom management for patients with cancer, the National Cancer Institute Cancer Moonshot Initiative funded the Improving the Management of symPtoms during And following Cancer Treatment (IMPACT) consortium. IMPACT includes 3 research centers that are implementing and testing systematic electronic symptom management systems (17). The Symptom Management IMplementation of Patient Reported Outcomes in Oncology (SIMPRO) is focused on building and testing an infrastructure for active symptom management. SIMPRO has deployed an electronic health record–integrated, patient-reported, outcome-based electronic symptom management (eSyM) program at 6 US health systems (4). The study protocol was approved by the Western Institutional Review Board and is registered on ClinicalTrials.gov (NCT03850912).

Initially, eSyM was set up to collect symptom reports from patients, using the past 7 days as the recall period (18). Early in the project, when clinicians called patients to address severe symptoms that had generated an alert, they observed that many of the symptoms had already resolved; therefore, those calls were perceived to be unnecessary and ineffective for active symptom support. To address this problem, a SIMPRO-wide change to shorten the recall period from the original “in the past 7 days” to “in the past 24 hours” was made (18). This retrospective analysis assessed the effects of that change in recall period on rates of reporting severe or moderate and severe symptoms.

### Data

eSyM assessments include 12 common cancer treatment–related symptoms and their presence (P), frequency (F), severity (S), and interference (I). Nine symptom terms (general pain [FSI], nausea [FS], vomiting [FS], constipation [S], shortness of breath [SI], fatigue [SI], anxiety [FSI], trouble drinking fluids [I], and poor appetite [SI]) are common in both chemotherapy and surgery versions. Three items each differ for the chemotherapy cohort (rash [P], numbness and tingling [SI], and diarrhea [F]) and surgery (wound redness [P], wound discharge [P], and painful urination [S]) versions of the assessment (Supplementary Table 2, available online). Patients could report up to 20 additional symptoms. The assessments were assigned for completion twice a week for up to 180 days for chemotherapy patients and 1-3 times per week for up to 2 months on a tapered schedule for surgery patients. Conditional branching logic guided the administration of symptom frequency, severity, and interference items. A composite symptom score was generated for each symptom using the Composite Grading Algorithm (19) for the PRO-CTCAE, which assigns scores to each individual item (ie, frequency, severity, and interference) and uses a prescribed algorithm to derive composite scores for each symptom using the individual item responses. Composite scores are expressed from 0 to 3, with 3 representing severe symptoms except for rash, wound discharge, and wound redness, which were reported as present or absent.

### Study population

The study included patients who responded to at least 1 eSyM assessment after initiation of cancer treatment or discharge after surgery. Patients with documented treatment plans for thoracic, gynecologic, or gastrointestinal cancers received the chemotherapy-specific assessment. Patients who had undergone a common surgical procedure to treat their disease received the surgery-specific assessment. Both versions were deployed in the Epic electronic health record patient portals (Epic Systems Corporation, Verona, WI). The stepped-wedge, cluster randomized trial design meant that eSyM was rolled out on a staggered schedule, with varying chemotherapy and surgery “go-live” dates for each SIMPRO practice.

*Interruption* was defined as the date the recall period changed from the past 7 days to the past 24 hours. It occurred after eSyM rollout, which was different for every SIMPRO practice (from July 2021 to April 2022). A 16-week window of observation (±8 weeks surrounding the interruption date) was created to standardize the analysis time horizon for surgery and chemotherapy cohorts at each SIMPRO practice. Creation of a 16-week window also allowed for a minimum of 8 observations before and after the interruption at most SIMPRO practices. Assessments were included in the window of observation only if they were submitted within 8 weeks of the interruption. Because of the staggered timing of the eSyM rollouts and interruption changes, practices lacking preinterruption data were excluded from analysis. Anonymized data were shared with Dana-Farber Cancer Institute, the coordinating center.

### Outcomes and predictors

The primary outcome was the proportion of completed assessments with 1 or more symptoms rated as severe (ie, composite score 3) across 12 common symptoms. Symptoms were aggregated weekly, with eight 7-day observation periods before and after the interruption. The secondary outcome was the proportion of assessments with 1 or more symptom reported as moderate or severe (ie, composite score 2 or 3) aggregated in a similar manner. For the primary outcome, proportions were calculated using the sum of assessments with at least 1 severe symptom as the numerator and the sum of all symptom assessments as the denominator. Similarly, for the secondary outcome, the sum of moderate or severe symptoms was included in the numerator and the sum of all symptom assessments as the denominator. Additionally, surveys reporting no symptoms (ie, score 0 for all items) were excluded from the analyses to assess the impact of the change in recall period on the rates of reporting severe symptoms among those respondents experiencing 1 or more symptoms. Also, symptom-specific analyses were performed for the 5 most prevalent symptoms: pain, fatigue, constipation, anxiety, and decreased appetite (20,21).

### Statistical analyses

Study variables were analyzed at a descriptive level. Patient characteristics were compared between those who responded within the 16-week window vs those who did not and separately between those who responded before vs after the interruption, using Wilcoxon rank-sum tests for continuous variables and Pearson χ^2^ or Fisher exact tests for categorical variables. Interrupted time series segmented regression analyses were applied to gauge the effects of a change in the recall period on rates of reporting severe symptoms across 12 common symptoms by fitting generalized linear mixed-effects models, with outcome modeled as a binomial distribution with the logit link function. We limited our analysis to assessing the coefficient of the interruption variable (ie, level change) to estimate the immediate effects of the recall period change. Variables included in the models were time before the interruption (‒8, ‒7, … , ‒1 week), time since the interruption (1, 2, … , 8 week), and interruption coded as an indicator variable. Patients were included as random effects to account for within-patient correlation. The analyses were performed for each SIMPRO practice separately, and point estimates, 95% confidence intervals (CIs), and *P* values for model coefficients were computed. The SIMPRO-wide effects of the interruption on rates of severe symptom reporting were calculated using the inverse variance weighting method commonly used in meta-analysis (22). Specifically, a weighted average of the log odds ratio (OR) estimates of the interruption was calculated, with each estimate weighted in inverse proportion to its variance. Separate models were run for the chemotherapy and surgery cohorts. Two-sided *P* values less than .05 was considered statistically significant. All statistical analyses were performed using R, version 4.2.1 (R Foundation for Statistical Computing, Vienna, Austria).

## Results

Overall, 7671 patients submitted at least 1 eSyM assessment between September 10, 2019, and June 30, 2022. The number of assessments per patient ranged from 1 to 32, with a median of 3 (interquartile range = 1-6). Of all eSyM respondents, 1692 (22%) patients completed 7823 eSyM assessments during the 16-week window of observation (±8 weeks from the interruption date). Briefly, the median age was 64 years (range = 55-72 years), 87% of respondents were White and 9% were Black in the window of observation. Gastroenterological cancers were the most prevalent diagnoses for chemotherapy (n = 366 [45%]) and gynecologic cancers were the most prevalent diagnoses for surgery patients (n = 370 [42%]). The characteristics of patients who responded during the window of observation, including body mass index, sex, ethnicity, employment, and marital status, were not statistically different from those who responded outside the window of observation. The proportion of patients from each of the 6 participating practices ranged from 7% to 29% (overall) and 7% to 34% in the preinterruption vs postinterruption cohorts (Table 1; Supplementary Table 1, available online). There were statistically significant differences in race, employment status, cancer type/surgery site, and SIMPRO practice between those who responded before the interruption vs after the interruption. Other characteristics of patients who responded after the interruption were not statistically different from those who responded before the interruption (Supplementary Table 1, available online). Considering staggered timing of the eSyM rollouts, differences in patient characteristics in the preinterruption vs postinterruption periods were expected.

**Table 1.** Characteristics of SIMPRO patients responding to at least 1 eSyM assessment, stratified by response time frame

Characteristic	Overall (N = 7671)	Included in the 16-wk window of observation		*P* ^a^
		Yes (n = 1692 [22%])	No (n = 5979 [78%])	
Age, median (IQR), y	64 (54-72)	64 (55-72)	63 (53-72)	.11
Race, No. (%)				.001
Asian	125 (1.6)	31 (1.8)	94 (1.6)	
Black	541 (7.1)	148 (8.7)	393 (6.6)	
White	6800 (89)	1469 (87)	5331 (89)	
Other^b^	111 (1.4)	32 (1.9)	79 (1.3)	
Unknown/declined	94 (1.2)	12 (0.7)	82 (1.4)	
Ethnicity, No. (%)				.85
Hispanic	151 (2.0)	36 (2.1)	115 (1.9)	
Non-Hispanic	7310 (95)	1611 (95)	5699 (95)	
Unknown	210 (2.7)	45 (2.7)	165 (2.8)	
Body mass index, No. (%)				.91
<25	2939 (38)	650 (38)	2289 (38)	
≥25	4732 (62)	1041 (62)	3690 (62)	
Unknown	1	1	0	
Sex, No. (%)				.10
Male	2634 (34)	553 (33)	2081 (35)	
Female	5037 (66)	1139 (67)	3898 (65)	
Marital status, No. (%)				.25
Married	4778 (62)	1070 (63)	3708 (62)	
Single	1238 (16)	288 (17)	950 (16)	
Divorced	785 (10)	160 (9.5)	625 (10)	
Widowed	631 (8.2)	122 (7.2)	509 (8.5)	
Other	239 (3.1)	52 (3.1)	187 (3.1)	
Employment status, No. (%)				.52
Employed	2579 (34)	578 (34)	2001 (33)	
Retired	3127 (41)	703 (42)	2424 (41)	
Disabled	591 (7.7)	117 (6.9)	474 (7.9)	
Unemployed	726 (9.5)	164 (9.7)	562 (9.4)	
Other	472 (6.2)	92 (5.4)	380 (6.4)	
Unknown	176 (2.3)	38 (2.2)	138 (2.3)	
Treatment modality, No. (%)				
Chemotherapy				.003
Gastrointestinal	1396 (43)	366 (45)	1030 (43)	
Gynecologic	586 (18)	160 (20)	426 (18)	
Thoracic	985 (31)	219 (27)	766 (32)	
Multi^c^	221 (6.8)	60 (7.4)	161 (6.7)	
Other	40 (1.2)	2 (0.2)	38 (1.6)	
Surgery				<.001
Gastrointestinal	1786 (40)	305 (34)	1481 (42)	
Gastrointestinal or gynecologic^d^	272 (6.1)	60 (6.8)	212 (5.9)	
Gynecologic	1384 (31)	370 (42)	1014 (29)	
Thoracic	811 (18)	106 (12)	705 (20)	
Unknown	190 (4.3)	44 (5.0)	146 (4.1)	
SIMPRO practice, No. (%)				<.001
Baptist Cancer Center	1707 (22)	404 (24)	1303 (22)	
Dana-Farber Cancer Institute	2235 (29)	570 (34)	1665 (20)	
Dartmouth Hitchcock Medical Center	1438 (19)	247 (15)	1191 (20)	
Lifespan Cancer Institute	582 (7.6)	177 (10)	405 (6.8)	
Maine Medical Center	522 (6.8)	119 (7.0)	403 (6.7)	
West Virginia University	1187 (15)	175 (10)	1012 (17)	

a Pearson χ^2^ test, Wilcoxon rank-sum test, Fisher exact test. SIMPRO = Symptom Management IMplementation of Patient Reported Outcomes in Oncology.

b “Other” includes respondents who selected “other,” 2 or more races, Native American/Alaska Native, or Native Hawaiian.

c More than 1 primary cancer site.

d Procedures performed for gastrointestinal or gynecologic cancers when unable to determine primary cancer by procedure code alone.

**Figure 1.**
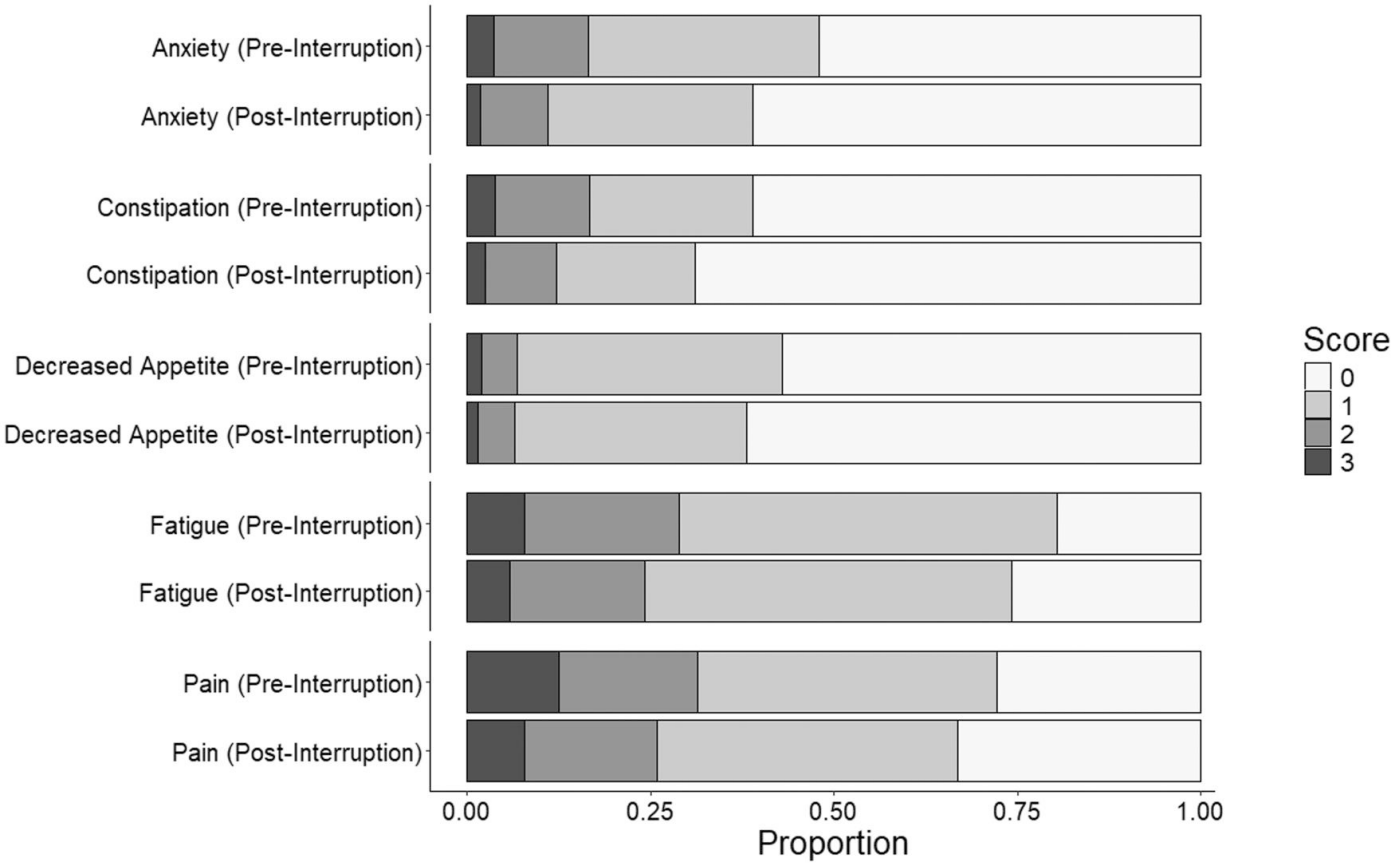
Proportions of the 5 most prevalent symptoms before and after interruption in the surgery cohort.

**Figure 2.**
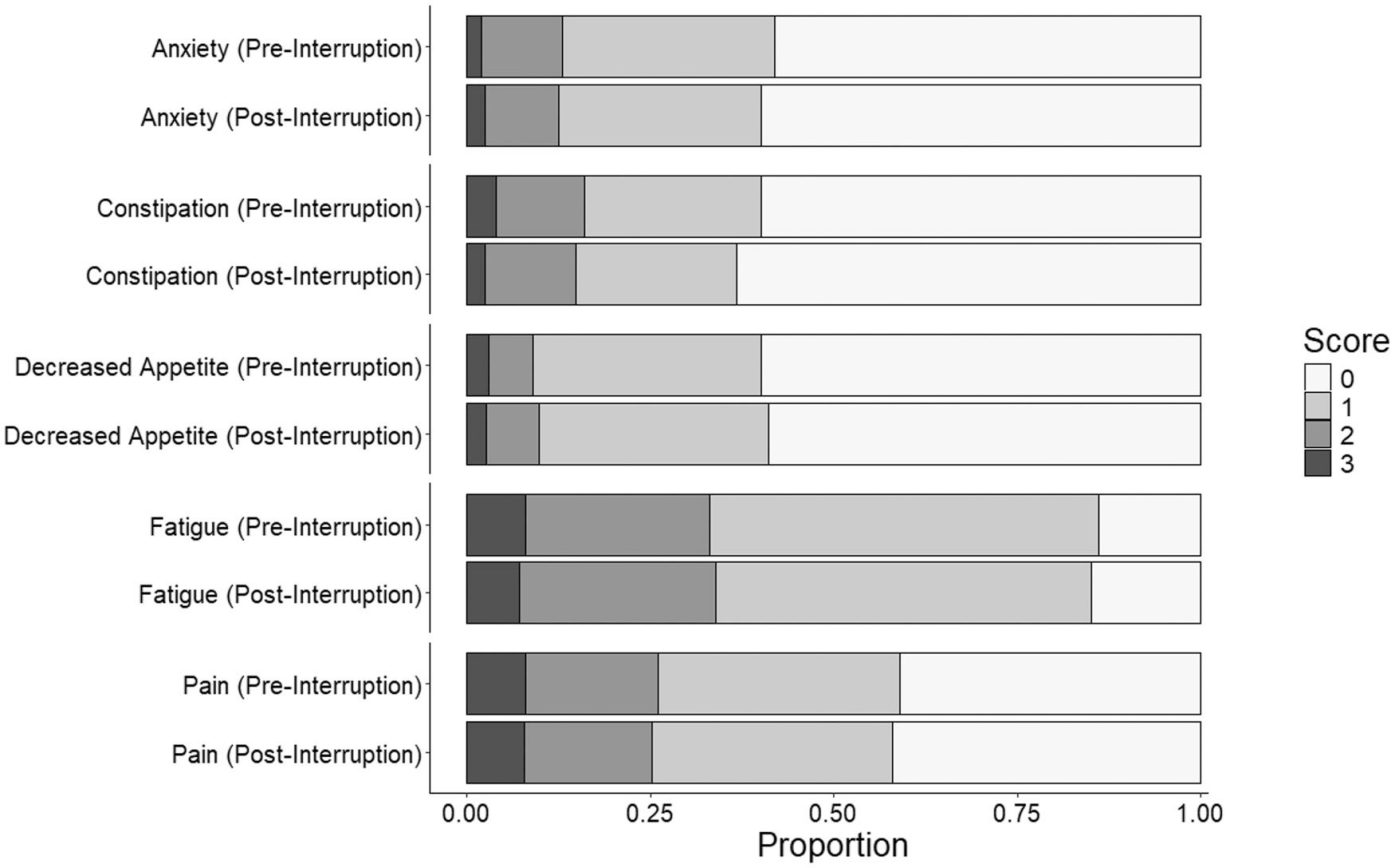
Proportions of the 5 most prevalent symptoms before and after interruption in the chemotherapy cohort.

At the SIMPRO consortium level, pain, fatigue, constipation, anxiety, and decreased appetite were the most frequently reported symptoms. Figures 1 and 2 display the distribution of scores for these 5 symptoms before and after interruption for the chemotherapy and surgery cohorts. The most prevalent symptoms were pain and fatigue, with most reporting mild (composite score 1) or moderate (composite score 2). Fewer than half of the respondents reported anxiety, constipation, or decreased appetite (Figures 1 and 2).

### Rates of reporting among surgery patients

The 24-hour recall period was associated with 35% lower odds of severe symptoms being reported in the surgery cohort (OR = 0.65, 95% CI = 0.46 to 0.93; *P *=* *.02), with symptom scores 0 to 3 included in the denominator. At the practice level, 3 of 4 surgical practices experienced lower odds of severe symptom reporting after changing the recall period, but the effect of the change was not statistically significant for the individual practices (Table 2). In the secondary analysis, the change in recall period was associated with 13% lower odds of reporting moderate or severe symptoms (OR = 0.87, 95% CI = 0.73 to 1.04; *P *=* *.14). At the practice level, the 24-hour recall period was associated with 33% lower odds of reporting moderate or severe symptoms at 1 of the 4 practices (OR = 0.67, 95% CI = 0.46 to 0.98; *P *=* *.04) (Table 3).

**Table 2.** Effects of recall period change on severe (composite score 3) symptom reporting consortium-wide and by SIMPRO practice^a^

	Odds ratio (95% confidence interval)	*P*
**Chemotherapy**		
SIMPRO Consortium	1.06 (0.85 to 1.33)	.60
Baptist Cancer Center	1.10 (0.69 to 1.74)	.70
Dana-Farber Cancer Institute	0.74 (0.41 to 1.35)	.32
Lifespan Cancer Institute	0.83 (0.26 to 2.68)	.76
Maine Medical Center	1.16 (0.55 to 2.45)	.69
**Surgery**		
SIMPRO Consortium	0.65 (0.46 to 0.93)	.02
Dana-Farber Cancer Institute	0.76 (0.46 to 1.27)	.30
Dartmouth Hitchcock Medical Center	0.58 (0.29 to 1.16)	.13
Lifespan Cancer Institute	1.15 (0.40 to 3.35)	.79
West Virginia University	0.54 (0.27 to 1.08)	.08

a SIMPRO = Symptom Management IMplementation of Patient Reported Outcomes in Oncology.

**Table 3.** Effects of recall period change on severe or moderate (composite score 2 or 3) symptom reporting consortium-wide and by SIMPRO practice^a^

	Odds ratio (95% confidence interval)	*P*
**Chemotherapy**		
SIMPRO Consortium	0.83 (0.71 to 0.97)	.02
Baptist Cancer Center	0.88 (0.68 to 1.12)	.29
Dana-Farber Cancer Institute	0.68 (0.53 to 0.92)	.01
Lifespan Cancer Institute	1.35 (0.79 to 2.30)	.27
Maine Medical Center	0.83 (0.60 to 1.14)	.26
**Surgery**		
SIMPRO Consortium	0.87 (0.73 to 1.04)	.14
Dana-Farber Cancer Institute	0.92 (0.70 to 1.20)	.54
Dartmouth Hitchcock Medical Center	0.67 (0.46 to 0.98)	.04
Lifespan Cancer Institute	1.08 (0.63 to 1.86)	.77
West Virginia University	0.93 (0.64 to 1.35)	.69

a SIMPRO = Symptom Management IMplementation of Patient Reported Outcomes in Oncology.

### Rates of reporting among chemotherapy patients

The 24-hour recall period was not associated with any significant differences in severe symptom reporting for the chemotherapy cohort (OR = 1.06, 95% CI = 0.85 to 1.33; *P *=* *.60) at the SIMPRO level or at the practice level. (Table 2). In the secondary analysis, there were 17% lower odds of moderate or severe symptom reporting (OR = 0.83, 95% CI = 0.71 to 0.97; *P *=* *.02). At the practice level, there were 32% lower odds of reporting moderate or severe symptom at 1 of the 4 practices (OR = 0.68, 95% CI = 0.53 to 0.92; *P *=* *.01) (Table 3).

### Additional analysis

First, we excluded symptoms reported as absent (ie, score 0) to determine whether changing the recall period increased the severity of symptom reporting among patients who reported nonzero symptoms. The 24-hour recall period was not associated with a difference in the likelihood of reporting more vs less severe symptoms for the chemotherapy cohort (OR = 1.07, 95% CI = 0.87 to 1.31; *P *=* *.51) or the surgery cohort (OR = 0.75, 95% CI = 0.53 to 1.06; *P *=* *.10) (Table 4). Second, we assessed the impact of the recall period on the 5 most prevalent symptoms (Table 5). Because relatively few patients reported moderate or severe scores for each individual symptom, the power to detect a significant effect at the symptom level was limited. The 24-hour recall period was associated with 49% lower odds of moderate or severe constipation among respondents undergoing surgical cancer treatment.

**Table 4.** Effects of recall period change on severe symptom (composite score 3) reporting consortium-wide and by SIMPRO practice (score 0 not included in the denominator)^a^

	Odds ratio (95% confidence interval)	*P*
**Chemotherapy**		
SIMPRO Consortium	1.07 (0.87 to 1.31)	.51
Baptist Cancer Center	1.09 (0.66 to 1.79)	.74
Dana-Farber Cancer Institute	0.78 (0.42 to 1.46)	.44
Lifespan Cancer Institute	0.78 (0.24 to 2.55)	.68
Maine Medical Center	1.13 (0.52 to 2.46)	.77
**Surgery**		
SIMPRO Consortium	0.75 (0.53 to 1.06)	.10
Dana-Farber Cancer Institute	0.82 (0.49 to 1.39)	.46
Dartmouth Hitchcock Medical Center	0.63 (0.30 to 1.31)	.21
Lifespan Cancer Institute	1.41 (0.45 to 4.41)	.55
West Virginia University	0.57 (0.28 to 1.18)	.13

a SIMPRO = Symptom Management IMplementation of Patient Reported Outcomes in Oncology.

**Table 5.** The odds of reporting severe and moderate to severe symptoms with a 24-hour vs 7-day recall for the 5 most prevalent symptoms^a^

	Severe symptoms		Moderate-severe symptoms	
	Odds ratio (95% confidence interval)	*P*	Odds ratio (95% confidence interval)	*P*
**Chemotherapy**				
Pain	1.55 (0.81 to 2.97)	.18	1.04 (0.96 to 1.12)	.37
Fatigue	0.65 (0.28 to 1.49)	.30	0.67 (0.44 to 1.01)	.06
Constipation	0.61 (0.15 to 2.48)	.49	0.83 (0.49 to 1.41)	.50
Anxiety	2.02 (0.30 to 13.95)	.47	1.59 (0.72 to 3.54)	.26
Decreased appetite	0.65 (0.22 to 1.98)	.45	1.00 (0.63 to 1.59)	.99
**Surgery**				
Pain	0.59 (0.30 to 1.14)	.12	0.77 (0.51 to 1.17)	.22
Fatigue	0.42 (0.17 to 1.06)	.07	0.89 (0.57 to 1.39)	.61
Constipation	0.27 (0.08 to 0.99)	.05	0.51 (0.31 to 0.85)	.01
Anxiety	0.78 (0.10 to 6.01)	.82	0.65 (0.31 to 1.37)	.26
Decreased appetite	0.99 (0.11 to 8.68)	.99	0.76 (0.29 to 2.00)	.58

a SIMPRO = Symptom Management IMplementation of Patient Reported Outcomes in Oncology.

## Discussion

In this analysis of real-world data derived from an electronic patient-reported outcomes system, after shortening the recall period from the “past 7 days” to the “past 24 hours,” we observed 35% lower odds of severe symptom reports following cancer surgeries. There was no significant difference in severe symptom reports among patients initiating chemotherapy, but the 24-hour recall period was associated with a 17% lower likelihood of reporting moderate or severe symptoms among those initiating chemotherapy. To our knowledge, this study is the first to evaluate the influence of differing recall periods on symptom prevalence rates, and it has important implications for implementing electronic patient-reported outcomes into routine practice.

Our findings suggest that a 24-hour recall period may fail to detect some severe symptoms among postoperative patients and some moderate to severe symptoms among chemotherapy patients. The 24-hour recall period may have helped focus attention on the symptoms that were bothersome at the moment and most amenable to clinical intervention. The differential effect of the recall period change on surgery vs chemotherapy patients was notable. Symptoms during the postoperative period, although potentially acute, may be less stable and more likely to resolve quickly. In contrast, symptoms among chemotherapy recipients can increase over time as the adverse effects of treatment build. In support of this theory, our prior work demonstrated that the frequency with which patients report severe symptoms quickly drops 2-3 weeks after surgery but remains relatively steady through 8 weeks after starting chemotherapy (20,21). Even among the chemotherapy cohort, however, we detected a modest decrease in the odds of moderate or severe symptom reporting after the recall period change. Our findings highlight the importance of considering how the recall period interacts with the symptom reporting interval. Shortening the recall period may have resulted in reduced reporting of symptoms, in part because patients were asked to report symptoms they had experienced only in the past 24 hours.

Prior studies have largely focused on comparatively assessing recall periods for patient-reported outcomes measures (9,14,23-25). For example, one study found that a 1-week recall period corresponded well with daily reporting, while acknowledging that a longer recall period could underestimate the true worst symptom experience among patients receiving chemotherapy or radiation therapy (9). When implementing electronic patient-reported outcomes for active symptom management, an alert that is proximal to the occurrence of a severe symptom is more actionable. A temporally distal symptom alert is analogous to a false-positive signal and could contribute to alert fatigue among the responding staff if the symptom had resolved. In prior studies, nurses reported receiving “too many” alerts (26) and raised concerns about alert fatigue (27). There is an urgent need to develop treatment pathways and algorithms to triage severe symptoms without burdening an overstretched workforce and compromising the sustainability of electronic patient-reported outcomes implementations (7).

Recall period and the frequency and timing of symptom assessment are related considerations. Their importance was demonstrated in a study that found that sparse, infrequent assessment resulted in underascertainment of treatment side effects across common symptoms (28). One downside of using a shorter recall period might be that symptoms occurring outside the recall period are missed. If symptom reports are intended to inform an efficacy or tolerability endpoint, this could introduce bias in interpreting clinical trials or real-world evidence. A recent study found that a 24-hour recall period had acceptable measurement properties for a clinical trials context when paired with daily reporting (16). Yet, among patients receiving treatment outside a clinical trial, daily reporting could be challenging to achieve, which raises a question about how frequently symptoms should be captured in routine practice and what the implications of the assessment interval and recall period may be on the analysis and interpretation of real-world symptom data (29,30). Additional evidence is needed to inform the design of patient-reported outcome–based symptom surveillance systems for use in clinical practice. The optimal recall period may vary based on clinical context, and the optimal assessment approach may vary by symptom. For example, symptoms that occur infrequently may require a longer recall period than those that persist or vary considerably from day to day (eg, insomnia) (9,23). Additionally, a longer recall period may not be suitable for patients with memory and cognitive impairment or in situations where symptoms are susceptible to recall bias (31).

This study has several limitations. First, temporal changes in practice patterns or patient populations could have affected severe symptom reporting unrelated to the change in recall period. At the same time, there were few differences in the participant characteristics of those whose reports were captured using the 7-day recall period vs the 24-hour recall period. To lessen the impact of secular trends, we used interrupted time series analysis to assess differences in rates of severe symptoms before and after interruption. Further, the eSyM rollout dates were randomized by practice and treatment modality, with varying dates over a 12-month period. These features make the study design less susceptible to secular trends and reduce confounding by lessening correlations with major events such as the COVID-19 pandemic (32). Second, respondents experienced relatively few severe symptoms, so our study design may have been underpowered to detect the effects of the interruption across all cohorts, institutions, symptoms, and time points. This effect could have explained why some subgroup and secondary analyses yielded mixed results. Third, eSyM requires patients to use a web-based portal. Our findings may not be generalizable to patients with limited access or proficiency with technology. Finally, our study looked at the impact of recall period on severe symptom reporting. Future analyses should assess the impact on clinically relevant outcomes, such as quality of life and emergency department utilization.

An interrupted time series analysis of the effects of shortening the recall period from the past 7 days to the past 24 hours for a set of 12 symptom-based electronic patient-reported outcome assessments resulted in a significant reduction in the odds of severe symptom reporting for surgery patients and a lesser but still statistically significant reduction in the odds of moderate or severe symptom reporting among chemotherapy patients. Our findings underscore the importance of considering electronic patient-reported outcome assessment intervals and recall period and the context of use.

## Supplementary Material

djae049_Supplementary_Data

## Data Availability

In accordance with Cancer Moonshot grant guidelines, data are to be publicly available once published. An online link is forthcoming, but until that portal is live, please send all requests for data as written proposals to the corresponding author.
