# Supplementary material for: Effects of a change in recall period on reporting severe symptoms: an analysis of a pragmatic multisite trial
Source: J Natl Cancer Inst. 2024 Mar 5;116(7):1137–44. doi: 10.1093/jnci/djae049 (PMC11223809; doi:10.1093/jnci/djae049)
Supplement: djae049_Supplementary_Data [file djae049_supplementary_data.pdf]

Supplementary Table 1. Pre- vs. Post-Interruption				
**Characteristic**	**Overall** N = 1,692	**Pre-Interruption** N = 978 (58%)	**Post-Interruption** N = 714 (42%)	**p-value**[1]
**Age** [Median (IQR)]	64 (55, 72)	64 (56, 72)	64 (53, 72)	0.17
**Race**				0.002
Asian	31 (1.8%)	16 (1.6%)	15 (2.1%)	
Black	148 (8.7%)	105 (11%)	43 (6%)	
Other	32 (1.9%)	12 (1.2%)	20 (2.8%)	
White	1,469 (87%)	838 (86%)	631 (88%)	
Unknown/Declined	12 (0.7)	7 (0.7)	5 (0.7)	
**BMI**				0.29
<25	650 (38%)	386 (40%)	264 (37%)	
>25	1,041 (62%)	591 (60%)	450 (63%)	
Unknown	1	1	0	
**Sex**				0.42
Male	553 (33%)	312 (32%)	241 (34%)	
Female	1,139 (67%)	666 (68%)	473 (66%)	
**Marital Status**				0.65
Married	1,070 (63%)	607 (62%)	463 (65%)	
Single	288 (17%)	175 (18%)	113 (16%)	
Divorced	160 (9.5%)	90 (9.2%)	70 (9.8%)	
Widowed	122 (7.2%)	75 (7.7%)	47 (6.6%)	
Other	52 (3.1%)	31 (3.2%)	21 (2.6%)	
**Ethnicity**				0.14
Non-Hispanic	1,611 (95%)	936 (96%)	675 (95%)	
Hispanic	36 (2.1%)	15 (1.5%)	21 (2.9%)	
Unknown	45 (2.7%)	27 (2.8%)	18 (2.6%)	
**Employment Status**				0.02
Employed	578 (34%)	312 (32%)	266 (37%)	
Retired	703 (42%)	413 (42%)	290 (41%)	
Disabled	117 (6.9%)	80 (8.2%)	37 (5.2%)	
Unemployed	164 (9.7%)	104 (11%)	60 (8.4%)	
Other	92 (5.4%)	46 (4.7%)	46 (6.4%)	
Unknown	38 (2.2%)	23 (2.4%)	15 (2.1%)	
**Treatment Modality**				
Chemotherapy				0.87
GI	366 (45%)	250 (45%)	116 (46%)	
THOR	219 (27%)	150 (27%)	69 (27%)	
GYN	160 (20%)	113 (20%)	47 (19%)	
Multi[2]	60 (7.4%)	39 (7.1%)	21 (8.3%)	
Other	2 (0.2%)	1 (0.2%)	1 (0.4%)	
Surgery				0.008

Supplementary Table 1. Pre- vs. Post-Interruption				
**Characteristic**	**Overall**<br>N = 1,692	**Pre-Interruption**<br>N = 978 (58%)	**Post-Interruption**<br>N = 714 (42%)	**p-value**[1]
GI	305 (34%)	131 (31%)	174 (38%)	
GYN	370 (42%)	204 (48%)	166 (36%)	
THORACIC	106 (12%)	48 (11%)	58 (13%)	
GI or GYN[3]	60 (6.8%)	26 (6.1%)	34 (7.4%)	
Other/Unknown	44 (5.0%)	16 (3.8%)	28 (6.1%)	
**SIMPRO Site**				<0.001
BAPT	404 (24%)	297 (30%)	107 (15%)	
DFCI	570 (34%)	292 (30%)	278 (39%)	
DHMC	247 (15%)	132 (13%)	115 (16%)	
LCI	177 (10%)	100 (10%)	77 (11%)	
MMC	119 (7%)	82 (8.4%)	37 (5.2%)	
WVU	175 (10%)	75 (7.7%)	100 (14%)	

[1]Pearson's Chi-squared test; Wilcoxon rank sum test; Fisher's exact test

[2] More than 1 primary cancer site

[3] Procedures performed for GI or GYN cancers, unable to determine primary cancer by procedure code alone

BAPT: Baptist Cancer Center

BMI: Body Mass Index

DFCI: Dana-Farber Cancer Institute

DHMC: Dartmouth-Hitchcock Medical Center

eSyM: Electronic Symptom Management Program

GI: Gastrointestinal

GYN: Gynecological

IQR: Interquartile Range

LCI: Lifespan Cancer Institute

MMC: Maine Medical Center

SIMPRO: Symptom Management Implementation of Patient-Reported Outcomes in Oncology

WVU: West Virginia University

Supplementary Table 2. Symptom Items

	**Pre-Interruption**<br>Recall period: In the past 7 days…		**Post-Interruption**<br>Recall period: In the past 24 hours…	
	**Chemotherapy**	**Surgery**	**Chemotherapy**	**Surgery**
**Core Symptoms**	Anxiety (FSI)<br>Constipation (S)<br>Fatigue (SI)<br>General Pain (FSI)<br>Poor Appetite (SI)<br>Nausea (FS)<br>Shortness of Breath (SI)<br>Trouble Drinking Fluids (I)**<br>Vomiting (FS)<br>Overall Wellbeing*<br>Physical Function*	Anxiety (FSI)<br>Constipation (S)<br>Fatigue (SI)<br>General Pain (FSI)<br>Poor Appetite (SI)<br>Nausea (FS)<br>Shortness of Breath (SI)<br>Trouble Drinking Fluids (I)*<br>Vomiting (FS)<br>Overall Wellbeing*<br>Physical Function*	Anxiety (FSI)<br>Constipation (S)<br>Fatigue (SI)<br>General Pain (FSI)<br>Poor Appetite (SI)<br>Nausea (FS)<br>Shortness of Breath (SI)<br>Trouble Drinking Fluids (I)*<br>Vomiting (FS)<br>Overall Wellbeing*<br>Physical Function*	Anxiety (FSI)<br>Constipation (S)<br>Fatigue (SI)<br>General Pain (FSI)<br>Poor Appetite (SI)<br>Nausea (FS)<br>Shortness of Breath (SI)<br>Trouble Drinking Fluids (I)**<br>Vomiting (FS)<br>Overall Wellbeing*<br>Physical Function*
**Additional symptoms**	Diarrhea (F)<br>Numbness and Tingling (SI)<br>Rash (P)	Painful Urination (S)<br>Wound Discharge (P)<br>Wound Redness (P)	Diarrhea (F)<br>Numbness and Tingling (SI)<br>Rash (P)	Painful Urination (S)<br>Wound Discharge (P)<br>Wound Redness (P)
**Assessment interval and duration**	Week 1 to Week 26<br>2 per week until 180 days unless series discontinued	Week 1 to Week 2<br>Three per week<br><br>Week 3-4<br>Two per week<br><br>Week 5-8<br>One per week	Week 1 to Week 26<br>2 per week until 180 days unless series discontinued	Week 1 to Week 2<br>Three per week<br><br>Week 3-4<br>Two per week<br><br>Week 5-8<br>One per week
**Response choices/anchors±**	Frequency (F):<br>Never (0)<br>Rarely (1)<br>Occasionally (2)<br>Frequently (3)<br>Almost constantly (4)	Severity (S):<br>None (0)<br>Mild (1)<br>Moderate (2)<br>Severe (3)<br>Very severe (4)	Interference (I):<br>Not at all (0)<br>A little bit (1)<br>Somewhat (2)<br>Quite a bit (3)<br>Very much (4)	
**Alert thresholds**	3	3	3	3

±Note: Scores of 3 or 4 are collapsed to score 3 (severe)
Note: F=Frequency; S=Severity; I=Interference
** Trouble drinking fluids – is adaptation of a PRO-CTCAE item
